# Better prediction of protein contact number using a support vector regression analysis of amino acid sequence

**DOI:** 10.1186/1471-2105-6-248

**Published:** 2005-10-13

**Authors:** Zheng Yuan

**Affiliations:** 1 Institute for Molecular Bioscience and ARC Centre in Bioinformatics, The University of Queensland, St. Lucia, 4072, Australia

## Abstract

**Background:**

Protein tertiary structure can be partly characterized via each amino acid's contact number measuring how residues are spatially arranged. The contact number of a residue in a folded protein is a measure of its exposure to the local environment, and is defined as the number of C_*β *_atoms in other residues within a sphere around the C_*β *_atom of the residue of interest. Contact number is partly conserved between protein folds and thus is useful for protein fold and structure prediction. In turn, each residue's contact number can be partially predicted from primary amino acid sequence, assisting tertiary fold analysis from sequence data. In this study, we provide a more accurate contact number prediction method from protein primary sequence.

**Results:**

We predict contact number from protein sequence using a novel support vector regression algorithm. Using protein local sequences with multiple sequence alignments (PSI-BLAST profiles), we demonstrate a correlation coefficient between predicted and observed contact numbers of 0.70, which outperforms previously achieved accuracies. Including additional information about sequence weight and amino acid composition further improves prediction accuracies significantly with the correlation coefficient reaching 0.73. If residues are classified as being either "contacted" or "non-contacted", the prediction accuracies are all greater than 77%, regardless of the choice of classification thresholds.

**Conclusion:**

The successful application of support vector regression to the prediction of protein contact number reported here, together with previous applications of this approach to the prediction of protein accessible surface area and B-factor profile, suggests that a support vector regression approach may be very useful for determining the structure-function relation between primary protein sequence and higher order consecutive protein structural and functional properties.

## Background

Prediction of protein three-dimensional structure from primary sequence is the central problem in structural bioinformatics. One approach is to use known structur∈homolog proteins as templates to determine the tertiary structures of novel proteins of unknown structure. Approaches include comparative modelling, threading and fold recognition methods. One protein structural feature is of particular interest here, namely, residue contact number (CN) which can be used to enhance protein fold recognition [1]. This measure has also been regarded as the conserved solvent exposure descriptor of similar folds without a common evolutionary origin [2]. Furthermore, contact number may be used to determine the energy function allowing molecular dynamics simulations of protein structures [3]. Here, we seek to use protein contact number to assist with the tertiary fold prediction of novel proteins for which an accurate functional relationship between a protein's primary sequence and its residues' contact numbers must be determined. To fulfil the task, we use a new method, the so-called support vector regression, to approximate the sequence-contact number relationship. We demonstrate that, as a result, we achieve more accurate predicted contact numbers than have been achieved to date.

The contact number, or coordination number, of a given residue of a folded protein is defined as the number of C_*β *_(or C_*α*_) atoms in other residues within a sphere around the C_*β *_(or C_*α*_) atom of that given residue. Previous approaches to the prediction of protein contact number fall into two categories: classification and regression. In the classification approach, residue contact numbers were first classified into two populations allowing a subsequent use of machine learning methods such as recurrent neural networks to perform predictions [4,5]. Unfortunately, decomposing contact numbers into two states via an arbitrary threshold oversimplifies the problem and much original information is lost. In contrast, the regression approach provides a direct and more accurate way to determine a functional relationship matching contact numbers and protein sequence and thus to provide more accurate contact number predictions. A recent study of Kinjo et al. [3], followed this approach but used a simple linear regression scheme to determine the functional relationship. They reported that the predicted and observe contact numbers had a correlation coefficient (CC) of 0.627. However, most functions in nature are non-linear and cannot be accurately approximated by linear formulas. Under the reasonable expectation that the sequence-contact number is indeed nonlinear, we use a more complicated machine learning method to determine the relationship and expect thereby to obtain more accurate predictions. In particular, we adopt a support vector regression (SVR) algorithm fully capable of determining a non-linear sequence-contact number relationship.

In our former work, we studied the dependence of protein accessible surface area (ASA) [6,7] and B-factor [8] on primary sequence. These works established that ASAs can be predicted and match observed values with a correlation coefficient of 0.69, while B-factors can be predicted and match observed values with a correlation coefficient of 0.53. These approaches established that multiple sequence inputs outperform single sequence inputs significantly. The importance of using multiple sequences was also observed in prior predictions of contact numbers [3]. Thus, in this present work, we focus on multiple sequence inputs. For completeness, we examine a range of different definitions of contact number ("consecutive" and "discrete"), and also examine whether including further information such as protein molecular weight and amino acid composition allows improved predictions. As a result, we are able to make predictions which match observed values with a correlation coefficient of 0.73, a significant improvement on earlier studies.

## Results

### Contact numbers according to different *r*_*d *_values

**Figure 1:**
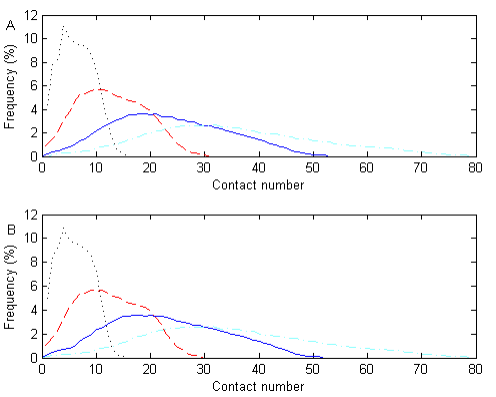
**Contact number distributions according to different definitions**. The radius cutoffs are selected as 8 Å, 10 Å, 12 Å and 14 Å, represented by dotted, slashed, solid and dot-and-slashed lines, respectively. A is for discrete contact number while B is for consecutive contact number.

We give 8 definitions of contact number and show their CN distributions in Fig. 1. For each definition, we compute the mean and standard deviation (Table 1). For the same radius cutoff *r*_*d*_, the discrete and consecutive definitions have very similar distributions with nearly the same mean and standard deviation. Their correlations are greater than 0.99 for all values of *r*_*d *_(8 Å, 10 Å, 12 Å and 14 Å). The contact numbers defined by different radius cutoffs have CCs greater than 0.83. Distributions with larger radius cutoffs more closely approximate normal distributions as their left-hand tails are almost all present. Since absolute contact numbers are normalized by a linear transformation (Equation 3), the general characteristics of their distributions will still be kept even after the normalization.

**Table 1:** The mean (N¯
 MathType@MTEF@5@5@+=feaafiart1ev1aaatCvAUfKttLearuWrP9MDH5MBPbIqV92AaeXatLxBI9gBaebbnrfifHhDYfgasaacH8akY=wiFfYdH8Gipec8Eeeu0xXdbba9frFj0=OqFfea0dXdd9vqai=hGuQ8kuc9pgc9s8qqaq=dirpe0xb9q8qiLsFr0=vr0=vr0dc8meaabaqaciaacaGaaeqabaqabeGadaaakeaadaqdaaqaaiabd6eaobaaaaa@2DE2@) and standard deviation (*SD*) of contact numbers according to different radius (*r*_*d*_) cutoffs. All results are expressed as (N¯
 MathType@MTEF@5@5@+=feaafiart1ev1aaatCvAUfKttLearuWrP9MDH5MBPbIqV92AaeXatLxBI9gBaebbnrfifHhDYfgasaacH8akY=wiFfYdH8Gipec8Eeeu0xXdbba9frFj0=OqFfea0dXdd9vqai=hGuQ8kuc9pgc9s8qqaq=dirpe0xb9q8qiLsFr0=vr0=vr0dc8meaabaqaciaacaGaaeqabaqabeGadaaakeaadaqdaaqaaiabd6eaobaaaaa@2DE2@, *SD*).

	*r*_*d *_= 8 Å	*r*_*d *_= 10 Å	*r*_*d *_= 12 Å	*r*_*d *_= 14 Å
Discrete	6.14, 3.29	12.90, 6.19	23.50, 10.46	35.39,15.39
Consecutive	6.27, 3.25	13.07, 6.14	23.56, 10.41	35.53, 15.39

**Figure 2:**
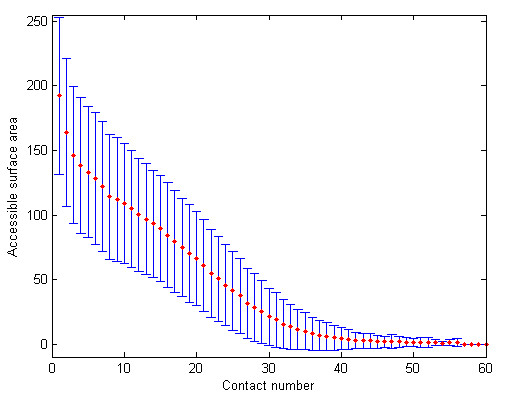
**The accessible surface area as a function of contact number**. Discrete contact numbers are used with a radius cutoff of 12 Å. Error bars represent the standard deviations.

To study the relationship between CN and ASA of a residue, we obtained the ASA for each residue in the 945 proteins using the DSSP program [9]. Using discrete definition of CN with a radius cutoff of 12 Å, we calculate the mean and standard deviation of ASA for each contact number and show the results in Fig. 2. A strong negative correlation between the two solvent exposure descriptors can be observed as indicated by a correlation coefficient of -0.734.

### Estimating the sequence-contact number relationship

When we train the SVR algorithm, normalized CNs are used instead of absolute CNs because the normalized values are always located between -3.0 and 3.0 for all radius cutoffs. Therefore, in all cases, the same set of SVR learning control parameters can be applied. Among the three groups of proteins (each has 315 chains), we estimate the sequence-contact number function in turn using one group and examine the estimated function using the remaining two groups. The correlation coefficient and root mean square error (RMSE) are computed for each test group, and their averages are shown in Table 2.

**Table 2:** Correlation coefficient (CC) and root mean square error (RMSE) for different contact number predictions. All results are expressed as mean ± standard deviation.

		*r*_*d *_= 8 Å	*r*_*d *_= 10 Å	*r*_*d *_= 12 Å	*r*_*d *_= 14 Å
Discrete	CC	0.64 ± 0.01	0.66 ± 0.01	0.69 ± 0.01	0.69 ± 0.01
	RMSE	0.77 ± 0.01	0.75 ± 0.01	0.72 ± 0.01	0.72 ± 0.02

Consecutive	CC	0.66 ± 0.01	0.67 ± 0.01	0.70 ± 0.01	0.70 ± 0.01
	RMSE	0.75 ± 0.01	0.74 ± 0.01	0.72 ± 0.01	0.72 ± 0.02

For all radius cutoffs, predictions using consecutive contact numbers are slightly better than predictions using their discrete counterparts. However, when the larger thresholds (e.g. 12 Å and 14 Å) are used, the accuracy difference decreases to insignificance. Previous work has shown that CNs with a radius cutoff of 12 Å or 14 Å are more useful for protein fold recognition [1]. Likewise, in this work, we also find that these cutoffs give better predictions. But, compared with the discrete contact numbers, the consecutive contact numbers give only a very slight improvement in predictions. The best accuracies are for consecutive contact numbers with thresholds of 12 Å and 14 Å, in which case the correlation coefficient between predicted and observed values can reach 0.70. If we convert the normalized contact numbers to their original absolute ones, the RMSE of 0.72 is equal to an actual error of 7.5 for a threshold of 12 Å, and an actual error of 11.1 for a threshold of 14 Å.

**Figure 3:**
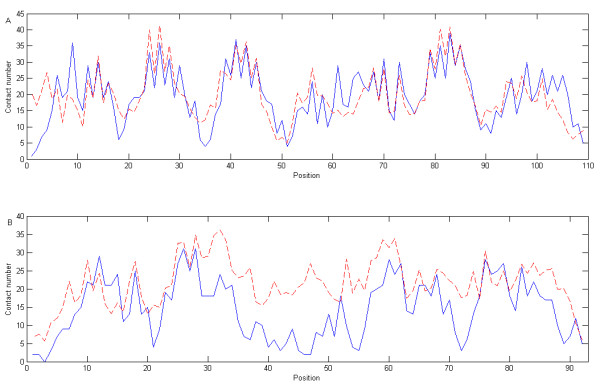
**The predicted and observed contact numbers for proteins GP130 (PDB: 1bj8) and Human chorionic gonadotropin (PDB: 1dz7, chain A)**. Discrete contact numbers are used with a radius cutoff of 12 Å. Observed and predicted contact numbers are represented by solid and dashed lines, respectively. A) GP 130 is predicted with a correlation coefficient of 0.75 and a root-mean-squar∈error of 6.07; B) Human chorionic gonadotropin is predicted with a correlation coefficient of 0.58 and a root mean square error of 9.73.

We also calculate the CC and RMSE for each individual protein using discrete contact numbers with a radius cutoff of 12 Å. The average CC and RMSE are then 0.67 and 7.31, respectively. More than half of the proteins are predicted with CCs greater than 0.70, and more than half are predicted with RMSEs less than 6.93. To illustrate the meaning of the CC and RMSE measures in this study, two comparisons of predicted and observed values are given in Figure 3. This figure shows the better agreement between the predicted and observed values in GP130 (PDB: 1bj8) as the CC is 0.75 and the RMSE is 6.07. In contrast, the prediction for human chorionic gonadotropin (PDB: 1dz7, chain A) yields a correlation coefficient of 0.58 and a RMSE of 9.73 with the region between position 40 and 50 being worst predicted.

### Sequence weight, as a feature, can improve prediction accuracy significantly

Prediction accuracy can be improved by taking account of protein size as measured by its weight. Given a sequence, the weight is the sum of individual weights of consisting amino acids. We use discrete contact numbers (radius cutoff = 12 Å) and divide all proteins into three groups with equal number of proteins, according to their weights. For the three groups, the weights (mean ± standard deviation) are 10611 ± 1637, 17421 ± 2666 and 40073 ± 5952 Daltons. Their average correlation coefficient between predicted and observed contact numbers are 0.61, 0.67 and 0.72, while their average RMSEs are 7.49, 7.09 and 7.35, respectively. These results suggest that smaller molecules are worst predicted.

To consider this effect, we calculate weight for each protein sequence and include this data as an additional input to the machine learning algorithm and re-run the training and testing procedures. Additional information, that of protein amino acid composition, was also included as an input, either individually or together with sequence weight data. For the separate groups of small chains, median chains, large chains and all chains, we calculate the mean and median values of the correlation coefficient between predicted and observed values of contact number, and their RMSEs, according to each set of different input information: local sequence ("LS"), local sequence plus sequence weight ("LS+W"), local sequence plus amino acid composition ("LS+AA") and local sequence plus sequence weight and amino acid composition ("LS+W+AA"). All results are shown in Table 3.

**Table 3:** Correlation coefficients (CCs) and root mean square errors (RMSEs) for individual proteins in different weight groups. The results are given as (mean, median).

		LS	LS+W	LS+AA	LS+W+AA
W≤3485	CC	0.61, 0.65	0.64, 0.67	0.62, 0.66	0.64, 0.67
	RMSE	7.49, 6.95	6.68, 6.41	7.24, 6.82	6.71, 6.45

13485<W≤22750	CC	0.67, 0.70	0.68, 0.71	0.68, 0.71	0.69, 0.72
	RMSE	7.09, 6.79	6.76, 6.54	7.05, 6.79	6.76, 6.60

W>22750	CC	0.72, 0.73	0.72, 0.74	0.72, 0.73	0.73, 0.74
	RMSE	7.35, 7.07	7.12, 6.95	7.24, 6.94	7.10, 6.90

All	CC	0.67, 0.70	0.68, 0.71	0.68, 0.71	0.68, 0.71
	RMSE	7.31, 6.94	6.86, 6.66	7.18, 6.86	6.86, 6.66

For all cases, it was determined that amino acid composition information can improve prediction performance. However, sequence weight can give yet more significant improvements. For example, in the group of small molecules, data about amino acid composition can increase the CC mean to 0.62 and decrease the RMSE mean to 7.24, while sequence weight data can increase the CC mean to 0.64 and decrease the RMSE mean to 6.68. When information about both sequence weight and amino acid composition is used together, we find still further improvement compared with using each data individually, although this may not be reflected by all measures. Particularly, the difference between "LS+W" and "LS+W+AA" is very minor. However, all the results clearly show that sequence weight is more important than amino acid composition in the prediction of contact numbers.

**Figure 4:**
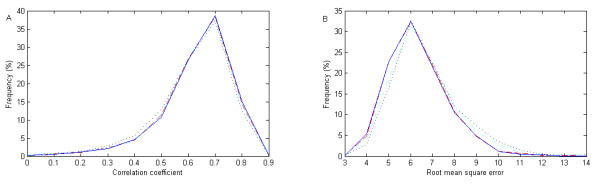
**Distributions of correlation coefficients and root mean square errors given different input information**. Discrete definition is used with a radius cutoff of 12 Å. The four inputs "LS", "LS+W", "LS+AA" and "LS+W+AA" are represented by dotted, slashed, dot-and-slashed and solid lines, respectively. A is for correlation coefficients while B is for root mean square errors.

Fig. 4 gives the overall distributions of CCs and RMSEs for 945 proteins. Compared with the CC, the RMSE is more sensitive in that its distribution more clearly reflects the improvement, while the distributions of "LS+W" and "LS+W+AA" are nearly identical. For all cases, the peak values of CC and RMSE are around 0.70 and 6.0, respectively.

In addition to the above analyses based on individual proteins, we also measure the accuracies on protein residues in the whole data set. We calculate CCs and normalized RMSEs for six testing groups, and express them as mean ± standard deviation, as given in Table 4. The CCs for "LS", "LS+W", "LS+AA" and "LS+W+AA" are 0.69, 0.733, 0.708 and 0.734, respectively. The normalized RMSEs are 0.72, 0.68, 0.71 and 0.68, respectively. Therefore, an obvious improvement can be found even if we measure it at the residue level.

**Table 4:** Correlation coefficients (CCs) and root mean square errors (RMSEs) are calculated for all residues when performing support vector regression algorithm using different input information.

	CC	RMSE
LS	0.69 ± 0.01	0.72 ± 0.01
LS+W	0.733 ± 0.005	0.68 ± 0.01
LS+AA	0.708 ± 0.009	0.71 ± 0.01
LS+W+AA	0.734 ± 0.006	0.68 ± 0.01

**Figure 5:**
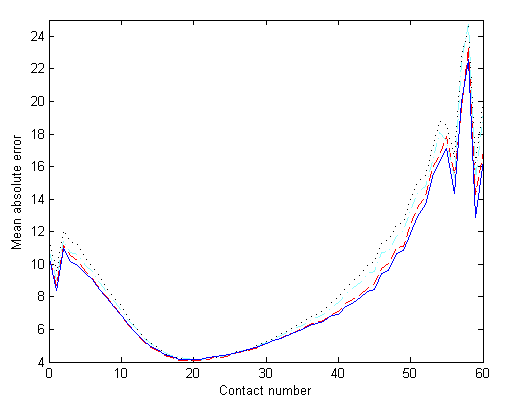
**The mean absolute errors for residues of different contact numbers**. The four inputs "LS", "LS+W", "LS+AA" and "LS+W+AA" are represented by dotted, slashed, dot-and-slashed and solid lines, respectively.

To measure the prediction performance for residues with different contact numbers, we compute the absolute errors for the residue with contact numbers from 0 to 60. The mean absolute errors for certain contact numbers are shown in Fig. 5, partitioned according to the four information inputs. Clearly, "LS+W+AA" gives the least absolute errors and therefore perform the best. Residues with about 20 contacting C_*β *_atoms are predicted with the least mean absolute error (4.1) and are the best predicted. This is because they have the largest number of samples in the dataset. Greater errors are found at each tail-end of the distribution corresponding to the residues with smaller or greater contact numbers. This is due to the small number of data points in each tail fed into support vector machines, and their representation is not adequate. Furthermore, this can be used to explain why the region from residue 40 to residue 50 of protein 1dz7 in Fig 3B is the worst predicted. This region mostly contains exposed residues with smaller contact numbers and thus, the residues cannot be well predicted by our method. An improvement on this part may be achieved by using other predicted features such as accessible surface area.

### Examining performance by formulating regression as a two-class problem

CN prediction has previously been examined as a two-class classification problem through use of a threshold with the accuracy being defined as the percentage of the correctly predicted residues on the overall residues [3-5]. However, this is not a good measure because the accuracy is susceptible to changes in the selected threshold that splits the data set. If the data set is heavily unbalanced, accuracy is always very high [10]. In this study, we use a different measure, and adopt the least (worst) prediction accuracy for each case to reflect its performance. Table 5 gives the least two-class prediction accuracies for eight definitions of contact number when using only local sequence information. Note that all the accuracies are the average of six tests. All accuracies are found to be greater than 74%, and in particular, when *r*_*d *_= 12 Å and a consecutive contact number definition is adopted, the least prediction accuracy is around 76.1%, which is comparable with the accuracy 76.3% recently reported based on choosing a particular threshold in linear models also using the same consecutive contact number definition [3].

**Table 5:** The least prediction accuracy (%) for two-class problems according to different contact number definitions. Only local sequence information is used.

	*r*_*d *_= 8 Å	*r*_*d *_= 10 Å	*r*_*d *_= 12 Å	*r*_*d *_= 14 Å
Discrete	74.1	74.5	75.8	75.2
Consecutive	75.6	75.7	76.1	75.6

**Figure 6:**
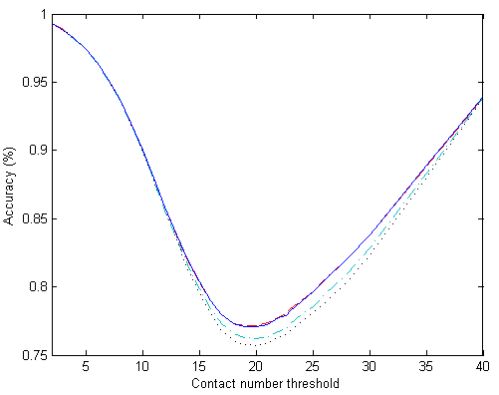
**Prediction accuracies when predictions are formulated as two-class problems using different contact number thresholds**. The four inputs "LS", "LS+W", "LS+AA" and "LS+W+AA" are represented by dotted, slashed, dot-and-slashed and solid lines, respectively.

We use discrete definitions of contact number and let *r*_*d *_= 12 Å. Using all the SVM outputs from six tests, we choose a number of thresholds to classify the data points as being either "contacted" or "non-contacted" and calculate their accuracies. All accuracies are plotted in Fig. 6, according to different information input. The least accuracies for "LS", "LS+W", "LS+AA" and "LS+W+AA" are 75.8%, 77.2%, 76.1% and 77.1%, respectively. Using sequence weight is much better than using amino acid composition.

## Discussion

Protein structural properties such as secondary structure, solvent accessibility and contact number provide valuable information for prediction of protein tertiary structures. How to improve the prediction accuracy of these parameters is still a challenging problem. Following Rost and Sander's pioneering work [11] on how to find a conserved and useful prediction index, Hamelryck [2] examined the conservation of nine solvent-exposure measures and found that contact number is the most conserved (correlation coefficient 0.72). His study suggested that CN is more suitable for fold recognition than other descriptors such as ASA. However, difficulties in accurately expressing the prediction problem (for example, it was previously framed as a two class problem using an arbitrary threshold) limited its further application. Recent work on contact number [3] formulating the problem for a regression analysis has enhanced studies in this area. From our work here, we confirm the utility of a regression analysis, and more specifically, establish that allowing for non-linearity via support vector regression allows a more accurate determination of the sequence-contact number relation which further illuminates relationships between protein structural and functional properties and their primary sequence and other features.

## Conclusion

We provide a new method for the prediction of protein contact number. Using protein local sequence information generated by multiple sequence alignments, the correlation coefficient between predicted and observed contact numbers can reach 0.70, with normalized root mean square error less than 0.72. The addition of information about sequence weight and amino acid composition as input features can increase the correlation coefficient to 0.734 and decrease the root mean square error to 0.68. This improvement is mainly attributed to the information about sequence weight while the information about amino acid composition only contributes slightly. Moreover, more than half of the proteins are predicted with correlation coefficients greater than 0.71. The prediction accuracies in the two-class problems, regardless of the cutoff thresholds, are greater than 77.0%. The successful application of SVR approach in this study suggests that it can more accurately describe the relationship between protein contact numbers and primary sequence.

## Methods

### Residue contact number

We take two definitions of contact number in this study, namely, that of "discrete" and "consecutive" contact number. The "discrete" contact number, *N*_*d*_, is defined by the number of C_*β *_atoms on other residues located within a sphere of radius *r*_*d *_centred on the C_*β *_atom of the residue of interest. The discrete contact number for *i*-th residue in a sequence with *M *residues is given by

Ndi=∑j:|j−i|>2Mσ(ri,j){σ(ri,j)=1ifri,j<rdσ(ri,j)=0ifri,j≥rd,     (1)
 MathType@MTEF@5@5@+=feaafiart1ev1aaatCvAUfKttLearuWrP9MDH5MBPbIqV92AaeXatLxBI9gBaebbnrfifHhDYfgasaacH8akY=wiFfYdH8Gipec8Eeeu0xXdbba9frFj0=OqFfea0dXdd9vqai=hGuQ8kuc9pgc9s8qqaq=dirpe0xb9q8qiLsFr0=vr0=vr0dc8meaabaqaciaacaGaaeqabaqabeGadaaakeaacqWGobGtdaqhaaqaaiabdsgaKbqaaiabdMgaPbaacqGH9aqpdaaeWbqaaGGaciab=n8aZjabcIcaOiabdkhaYnaaBaaaleaacqWGPbqAcqGGSaalcqWGQbGAaeqaaOGaeiykaKIaemiiaaIaemiiaaIaemiiaaIaemiiaaIaemiiaaIaemiiaaIaemiiaaIaemiiaaIaemiiaaIaemiiaaIaemiiaaIaemiiaaYaaiqaaeaafaqabeGabaaabaGae83WdmNaeiikaGIaemOCai3aaSbaaSqaaiabdMgaPjabcYcaSiabdQgaQbqabaGccqGGPaqkcqGH9aqpcqaIXaqmcqWGGaaicqWGGaaicqqGPbqAcqqGMbGzcqWGGaaicqWGYbGCdaWgaaWcbaGaemyAaKMaeiilaWIaemOAaOgabeaakiabgYda8iabdkhaYnaaBaaaleaacqWGKbazaeqaaaGcbaGae83WdmNaeiikaGIaemOCai3aaSbaaSqaaiabdMgaPjabcYcaSiabdQgaQbqabaGccqGGPaqkcqGH9aqpcqaIWaamcqWGGaaicqWGGaaicqqGPbqAcqqGMbGzcqWGGaaicqWGYbGCdaWgaaWcbaGaemyAaKMaeiilaWIaemOAaOgabeaakiabgwMiZkabdkhaYnaaBaaaleaacqWGKbazaeqaaaaaaOGaay5EaaGaeiilaWIaaCzcaiaaxMaadaqadaqaaiabigdaXaGaayjkaiaawMcaaaqaaiabdQgaQjabdQda6iabdYha8jabdQgaQjabgkHiTiabdMgaPjabdYha8jabg6da+iabikdaYaqaaiabd2eanbGaeyyeIuoaaaa@8A4A@

where *r*_*i*,*j *_is the distance between the C_*β *_atoms of the *i*th and *j*th residues which are understood to be separated in sequence by at least two amino acids. Note that Ndi
 MathType@MTEF@5@5@+=feaafiart1ev1aaatCvAUfKttLearuWrP9MDH5MBPbIqV92AaeXatLxBI9gBaebbnrfifHhDYfgasaacH8akY=wiFfYdH8Gipec8Eeeu0xXdbba9frFj0=OqFfea0dXdd9vqai=hGuQ8kuc9pgc9s8qqaq=dirpe0xb9q8qiLsFr0=vr0=vr0dc8meaabaqaciaacaGaaeqabaqabeGadaaakeaacqWGobGtdaqhaaWcbaGaemizaqgabaGaemyAaKgaaaaa@30AA@ is a discrete integer. By replacing the step function *σ*(*r*_*i*,*j*_) with a sigmoid function, Ndi
 MathType@MTEF@5@5@+=feaafiart1ev1aaatCvAUfKttLearuWrP9MDH5MBPbIqV92AaeXatLxBI9gBaebbnrfifHhDYfgasaacH8akY=wiFfYdH8Gipec8Eeeu0xXdbba9frFj0=OqFfea0dXdd9vqai=hGuQ8kuc9pgc9s8qqaq=dirpe0xb9q8qiLsFr0=vr0=vr0dc8meaabaqaciaacaGaaeqabaqabeGadaaakeaacqWGobGtdaqhaaWcbaGaemizaqgabaGaemyAaKgaaaaa@30AA@ becomes a real number. This procedure was previously adopted by Kinjo et al. [3] to smooth the discrete contact numbers. A particular sigmoid function is given by

*σ*(*r*_*i*,*j*_) = 1/{1 + exp [3(*r*_*i*,*j *_- *r*_*d*_)]}.     (2)

We have tried four values of *r*_*d *_(8 Å, 10 Å, 12 Å and 14 Å) with discrete and consecutive definitions and thus have 8 combinations all of which will be used in our SVR approach.

### Normalization of contact number

The distributions of contact numbers can be approximated by normal distributions, as shown in Fig. 1. With respect to a certain *r*_*d*_, we calculate the mean (N¯
 MathType@MTEF@5@5@+=feaafiart1ev1aaatCvAUfKttLearuWrP9MDH5MBPbIqV92AaeXatLxBI9gBaebbnrfifHhDYfgasaacH8akY=wiFfYdH8Gipec8Eeeu0xXdbba9frFj0=OqFfea0dXdd9vqai=hGuQ8kuc9pgc9s8qqaq=dirpe0xb9q8qiLsFr0=vr0=vr0dc8meaabaqaciaacaGaaeqabaqabeGadaaakeaadaqdaaqaaiabd6eaobaaaaa@2DE2@) and standard deviation (*SD*). So, the normalized contact number *N*_*norm *_is determined by the following formula:

Nnorm=N−N¯SD.     (3)
 MathType@MTEF@5@5@+=feaafiart1ev1aaatCvAUfKttLearuWrP9MDH5MBPbIqV92AaeXatLxBI9gBaebbnrfifHhDYfgasaacH8akY=wiFfYdH8Gipec8Eeeu0xXdbba9frFj0=OqFfea0dXdd9vqai=hGuQ8kuc9pgc9s8qqaq=dirpe0xb9q8qiLsFr0=vr0=vr0dc8meaabaqaciaacaGaaeqabaqabeGadaaakeaacqWGobGtdaWgaaWcbaGaemOBa4Maem4Ba8MaemOCaiNaemyBa0gabeaakiabg2da9maalaaabaGaemOta4KaeyOeI0Yaa0aaaeaacqWGobGtaaaabaGaem4uamLaemiraqeaaiabc6caUiaaxMaacaWLjaWaaeWaaeaacqaIZaWmaiaawIcacaGLPaaaaaa@3EE6@

At the first step, we predict the normalized contact number because 1) it is easy to handle the data, and 2) it is easy to compare the results for different *r*_*d *_thresholds. At the second step, we recover the absolute contact numbers from their predicted normalized values using this equation.

### Sequence coding

We predict contact number from protein local sequence. For a given residue, the local sequence contains its N-terminal and C-terminal seven nearest-neighbour residues. Thus, the local sequence makes a window of fifteen amino acids. We code each residue in the window using the PSI-BLAST position-specific scoring matrix [12]. The matrices are obtained by querying the input sequence using PSI-BLAST against the NCBI non-redundant protein sequence database with three rounds, masking coil-coiled and low-complexity regions [13]. The elements in the row of the matrix reflect the probabilities for 20 amino acids occurring at this position. All the elements are divided by 10 for normalization and thus each residue is represented by a 20-dimesional vector. Since the residues in coil-coil and low-complexity regions do not have meaningful scores, we encode the residue with an orthogonal scheme. In the 20-dimensional vector coding a given residue, only the entry representing this type of amino acid is assigned as 0.5 with all other entries set as zeros. To consider the terminal residues, we expend the 20-dimensional vector to being 21-dimensional for all residues. When the last entry is set as 0.5 and other entries have zeros, it represents a blank residue added to the N-terminal or the C-terminal to make a local sequence of 15-residue length. For all other residues, the 21-st entries are set to zero. In summery, a residue is coded by a 315-dimensional vector.

### Support vector regression

To find the function between protein local sequence and normalized contact number, we use ∈-insensitive support vector regression (∈-SVR) [14,15]. The expected function can be formulated as

*f*(*X*_*i*_) = 〈*W*, Φ(*X*_*i*_)〉 + *b*,     (4)

where *W *is the weight and *b *is the bias. Φ(*X*_*i*_) is a non-linear function mapping a data point from the input space to the feature space, so consequently, SVR is able to perform non-linear regression. The goal of the regression is to find the optimal *W *and *b *using some optimisation criteria. In ε-SVR, errors greater than ε are penalized, where two positive variables *ξ *and ξ* are used to measure the deviation of samples outside the *ε*-insensitive tube. The optimisation problem can be expressed as

Minimize12‖W‖2+C∑i=1M(ξi+ξi*),
 MathType@MTEF@5@5@+=feaafiart1ev1aaatCvAUfKttLearuWrP9MDH5MBPbIqV92AaeXatLxBI9gBaebbnrfifHhDYfgasaacH8akY=wiFfYdH8Gipec8Eeeu0xXdbba9frFj0=OqFfea0dXdd9vqai=hGuQ8kuc9pgc9s8qqaq=dirpe0xb9q8qiLsFr0=vr0=vr0dc8meaabaqaciaacaGaaeqabaqabeGadaaakeGabaa6imXvP5wqSXMqHnxAJn0BKvguHDwzZbqegyvzYrwyUfgaiqaacaWFnbGaa8xAaiaa=5gacaWFPbGaa8xBaiaa=LgacaWF6bGaa8xzaiaaxMaadaWcaaqaaiabigdaXaqaaiabikdaYaaadaqbdaqaaiabdEfaxbGaayzcSlaawQa7amaaCaaaleqabaGaeGOmaidaaOGaey4kaSIaem4qam0aaabCaeaacqGGOaakiiGacqGF+oaEdaWgaaWcbaGaemyAaKgabeaakiabgUcaRiab+57a4naaDaaaleaacqWGPbqAaeaacqGGQaGkaaGccqGGPaqkaSqaaiabdMgaPjabg2da9iabigdaXaqaaiabd2eanbqdcqGHris5aOGaeiilaWcaaa@5B39@

subject  to{f(Xi)−yi≤ε+ξiyi−f(Xi)≤ε+ξi*ξi,ξi*≥0  for i=1,…,M     (5)
 MathType@MTEF@5@5@+=feaafiart1ev1aaatCvAUfKttLearuWrP9MDH5MBPbIqV92AaeXatLxBI9gBaebbnrfifHhDYfgasaacH8akY=wiFfYdH8Gipec8Eeeu0xXdbba9frFj0=OqFfea0dXdd9vqai=hGuQ8kuc9pgc9s8qqaq=dirpe0xb9q8qiLsFr0=vr0=vr0dc8meaabaqaciaacaGaaeqabaqabeGadaaakeGabaa6imXvP5wqSXMqHnxAJn0BKvguHDwzZbqegyvzYrwyUfgaiqaacaWFZbGaa8xDaiaa=jgacaWFQbGaa8xzaiaa=ngacaWF0bGaaGPaVlaaykW7caWF0bGaa83BaiaaxMaadaGabaqaauaabeqadeaaaeaacqWGMbGzcqGGOaakcqWGybawdaWgaaWcbaGaemyAaKgabeaakiabcMcaPiabgkHiTiabdMha5naaBaaaleaacqWGPbqAaeqaaOGaeyizImkcciGae4xTduMaey4kaSIae4NVdG3aaSbaaSqaaiabdMgaPbqabaaakeaacqWG5bqEdaWgaaWcbaGaemyAaKgabeaakiabgkHiTiabdAgaMjabcIcaOiabdIfaynaaBaaaleaacqWGPbqAaeqaaOGaeiykaKIaeyizImQae4xTduMaey4kaSIae4NVdG3aa0baaSqaaiabdMgaPbqaaiabcQcaQaaaaOqaaiab+57a4naaBaaaleaacqWGPbqAaeqaaOGaeiilaWIae4NVdG3aa0baaSqaaiabdMgaPbqaaiabcQcaQaaakiabgwMiZkabicdaWiabbccaGiabbccaGiabbAgaMjabb+gaVjabbkhaYjabbccaGiabdMgaPjabg2da9iabigdaXiabcYcaSiablAciljabcYcaSiabd2eanbaaaiaawUhaaiaaxMaacaWLjaWaaeWaaeaacqaI1aqnaiaawIcacaGLPaaaaaa@8590@

where *C *is the regularization constant that determines the trad∈off between the norm and the error penalty.

The solution of the above problem was given by the authors of ∈SVR [14,15] as follows,

f(X)=∑i=1M(αi−αi*)〈Φ(Xi),Φ(X)〉+b,     (6)
 MathType@MTEF@5@5@+=feaafiart1ev1aaatCvAUfKttLearuWrP9MDH5MBPbIqV92AaeXatLxBI9gBaebbnrfifHhDYfgasaacH8akY=wiFfYdH8Gipec8Eeeu0xXdbba9frFj0=OqFfea0dXdd9vqai=hGuQ8kuc9pgc9s8qqaq=dirpe0xb9q8qiLsFr0=vr0=vr0dc8meaabaqaciaacaGaaeqabaqabeGadaaakeaacqWGMbGzcqGGOaakcqWGybawcqGGPaqkcqGH9aqpdaaeWbqaaiabcIcaOGGaciab=f7aHnaaBaaaleaacqWGPbqAaeqaaOGaeyOeI0Iae8xSde2aa0baaSqaaiabdMgaPbqaaiabcQcaQaaakiabcMcaPmaaamaabaGaeuOPdyKaeiikaGIaemiwaG1aaSbaaSqaaiabdMgaPbqabaGccqGGPaqkcqGGSaalcqqHMoGrcqGGOaakcqWGybawcqGGPaqkaiaawMYicaGLQmcacqGHRaWkcqWGIbGycqGGSaalcaWLjaGaaCzcamaabmaabaGaeGOnaydacaGLOaGaayzkaaaaleaacqWGPbqAcqGH9aqpcqaIXaqmaeaacqWGnbqta0GaeyyeIuoaaaa@5667@

where *α*_*i *_and αi*
 MathType@MTEF@5@5@+=feaafiart1ev1aaatCvAUfKttLearuWrP9MDH5MBPbIqV92AaeXatLxBI9gBaebbnrfifHhDYfgasaacH8akY=wiFfYdH8Gipec8Eeeu0xXdbba9frFj0=OqFfea0dXdd9vqai=hGuQ8kuc9pgc9s8qqaq=dirpe0xb9q8qiLsFr0=vr0=vr0dc8meaabaqaciaacaGaaeqabaqabeGadaaakeaaiiGacqWFXoqydaqhaaWcbaGaemyAaKgabaGaeiOkaOcaaaaa@30B6@ are Lagrange multipliers. We can replace 〈Φ(*X*_*i*_), Φ(*X*)〉, the inner product of Φ(*X*_*i*_) and Φ(*X*), by a kernel function *K*(*X*_*i*_, *X*), if *K*(*X*_*i*_, *X*) = 〈Φ(*X*_*i*_), Φ(*X*)〉. The radial basis function are used in our study, as given by

*K*(*X*_*i*_, *X*) = exp(-*γ*||*X*_*i *_- *X*||^2^),     (7)

where *γ *is a parameter to be tuned by the user.

We constantly set ε as 0.01, *γ *as 0.01 and *C* as 5.0, because this set of parameters yielded the best performance in our previous work [6,8]. A number of software packages can be used to find the solution such as SVMlight [16].

### Dataset preparation and prediction evaluation

To test our approach, we selected 945 unique protein chains, which were previously used for prediction of protein ASA, and were prepared by PDB-REPRDB [17]. The structures solved by X-ray crystallography were with resolution less than 2.0 Å and with an R-factor less than 0.2. All chains are at least 60 amino acids or longer, and the pair-wise identity is less than 25%. The protein names can be found in the additional file 1 (supplementary material).

The proteins are randomly divided into three groups with each group having 315 chains. Each group is in turn used for training with the remaining two groups used for testing. Therefore, each group is tested twice by the two functions derived from the other groups, and as a result we have six groups of examination results.

Pearson's correlation coefficients and root mean square errors are calculated with respects to all residues and individual proteins. In addition, the absolute errors are calculated for the residues with different contact numbers. In order to compare with previous classification methods, we use different thresholds to classify contact numbers as "contacted" or "non-contacted" and compute the overall accuracy. The accuracy is defined as the ratio between the number of correctly predicted residues and the total number.

## Supplementary Material

Additional File 1 **The names of 945 protein chains**. The first four characters are their PDB names. The fifth is the chain name and "_" means single chain. Click here for file
