# Supplementary material for: Better prediction of protein contact number using a support vector regression analysis of amino acid sequence
Source: BMC Bioinformatics. 2005 Oct 13;6:248. doi: 10.1186/1471-2105-6-248 (PMC1277819; doi:10.1186/1471-2105-6-248)
Supplement: Additional File 1 — The names of 945 protein chains. The first four characters are their PDB names. The fifth is the chain name and "_" means single chain. [file 1471-2105-6-248-S1.doc]

945 protein chains used for evaluation of support vector regression algorithms

| Group 1 | Group 2 | Group 3 |
| --- | --- | --- |
| 1bqv_  1jh6a  1fil_  1o0la  1acf_  1fipa  1ngla  1cg7a  1k12a  1jaja  1eq1a  1gd0a  1kufa  1hzta  1d9ca  1b8aa  1ji8a  2pgd_  1uaqa  1dsba  1o78a  1ajz_  1qcka  1h5pa  1pls_  1pce_  1cqya  1zug_  1icha  1ji1a  1bj8_  1gdea  2ezl_  1khma  1qkla  1oqcb  1c01a  1o8pa  1bm8_  1a4ua  1xpa_  1mwya  1agi_  1ccza  1az9_  1m42a  1ghta  1g61a  1cewi  1cdy_  1dk8a  1vcaa  1nepa  1gxya  1lj5a  1mla_  1qtwa  1fn9a  1c05a  1ivta  1j8qa  1gpr_  1ieza  1fwp_  1jbma  1kfra  1e39a  1jm7b  1kkxa  1ju2a  1inlc  1m8la  2sqca  1gjsa  1fsfa  1f16a  1qcxa  1ex2a  1jdqa  1dqia  2vik_  1a8h_  1ldsa  1jxsa  1e3aa  1a44_  1j9ia  1bkpb  1iqqa  2fmr_  1g7oa  1m55a  1eiwa  1j33a  2ezi_  1ako_  1gl5a  1kvea  1qnra  1fp3a  1jw3a  1g90a  1b8ta  1cem_  1brt_  1enwa  1o0wb  1k0xa  1kn7a  1qtsa  1emwa  153l_  2sak_  1l7ya  1bgf_  1a7ta  1fj7a  1ewwa  1fpzc  1mgta  1ew0a  1byma  1ceja  1edt_  1tvs_  1d1da  2abh_  2tnfa  1kvna  1jh3a  1g2qa  1ehxa  1g6za  1kn6a  2scpa  1c20a  17gsa  1cdq_  1hs7a  1j7qa  1nah_  1dhma  1nwpa  1n89a  1e30a  1k99a  1k19a  1fw9a  2tmp_  1lea_  2fcba  1cc8a  1huua  1iaza  1fc9a  1ispa  2baa_  1m62a  1irf_  1pmi_  1msc_  1klxa  1iooa  1m39a  1ht9a  1li1a  1g25a  1dgna  1n2za  1pcfa  1i5ja  1jfla  1svb_  1eyvb  1ckv_  1gxqa  1fo5a  1qi7a  1g8qa  1io0a  1qmya  1aoca  1tfb_  2i1b_  1f46b  1cpn_  1a6s_  1dqba  2naca  1m94a  1sxl_  1jt8a  1l0gb  1jyha  1ehga  1fht_  1ds0a  1tbd_  1lxl_  1qaza  1cfb_  1aab_  1mtyd  1ksr_  1lxza  1o8ra  1kkja  1itf_  1arb_  2if1_  1ixda  3grs_  1hfc_  1apyb  1igs_  1hi7a  1d9na  1bbi_  1nlbh  1idpa  1h8ma  1jwea  1df3a  1mn8d  1ijaa  1qtta  1itpa  1gof_  1qqva  1a8l_  1iq3a  1c9qa  1gnya  1bw3_  1f5va  1nct_  1hb6a  4ubpb  3chy_  1koe_  1cqxa  1adoa  1ltsa  1sra_  1f0za  1i2ea  1tns_  1bqk_  1irza  1joya  1fhoa  1g7ea  1vpnb  1dj0a  1jwwa  1gcg_  1ey4a  1msk_  1or7c  1gh8a  2cpl_  1j0ta  1duga  1de3a  2utga  1opd_  1iyga  1l7aa  1qksa  1j4ga  2ifea  1svfa  1b5qb  1cxpa  1mtyb  1bhu_  1qgia  1mml_  1aiw_  1kkda  1pcn_  1ib8a  1yge_  1hsq_  1koya  1fp0a  1c7ia  1rzl_  1ck2a  1bt7_  2rn2_  2spca  1i6fa  1by1a  1fc3a  1b3aa  1bbn_  1tpg_  1b2pa  1dbda  1esia  1dfx_  1sur_  1thfd  1nar_  1jlna  1h6ta  1n1ja  1n3ga  1gnc_  1ly2a  1gjxa  1dz7a  1nyna  1b75a | 1chc_  1mixa  1a3aa  1qd9a  1i9ga  1j3ga  1f5xa  1kbla  1hf8a  1jl7a  1kdgb  1pud_  1yaca  1hd0a  1fafa  1qu5a  2ptd_  2gdm_  1tit_  1pms_  1muga  1dipa  1ivua  1hre_  1ft5a  1bn8a  1kzqa  1k0ha  1i2sa  1bxaa  1irda  1hpwa  1ilya  1dgwa  1eika  2nef_  2rgf_  1ktma  1esgb  1cpcb  1h4ia  1sfta  1rip_  1rro_  1h40a  1ks8a  2lfb_  1bu7a  1nbca  1dtva  1l8ca  1faza  1l3ha  1kkga  1bhta  1ig6a  1xnb_  1cc4a  1cxwa  1unka  1gcqc  1gybb  1fgp_  1h03p  1msza  1duwa  1bb8_  1iufa  1trla  1b64_  1orb_  1dysa  1cvra  1tib_  1lre_  1iiba  1dx8a  1a68_  1k5wa  1du2a  1qnf_  1k20a  1kola  1jk7a  1g6ea  1iioa  1h0la  1e3ya  1cvo_  1vhh_  1tbc_  1lbwa  1k1ga  1f5ya  1l5ca  1eoka  2end_  1ftra  1xgsa  1dgwy  1bslb  1gyza  1krs_  1gkna  1kq3a  3mspa  2adr_  1bfg_  1klqa  1f2ha  1dvoa  1dz1a  1i35a  1prr_  1dgwx  1bola  1lq9a  1l6wa  1h6qa  1m44b  1iba_  2gcc_  1b5ea  1pbn_  1abv_  1h6ha  1hcd_  1iab_  1ah7_  4ubpa  1o1wa  1cmba  1cih_  1k7xb  1exg_  1oa4a  4srna  1mut_  1e68a  1bq0_  1saca  1dqga  1ifwa  1ireb  2hvm_  1mk0a  1kepa  1agja  1o0ya  1af7_  1bkza  2qwa_  1fbr_  1g10a  1dmha  1itya  1regx  1ej5a  1aci_  1dhn_  1npk_  1qu6a  1gh9a  1jdc_  1aqb_  1elka  1fwqa  1k5ka  2cmd_  1eo1a  1who_  1clh_  1tmy_  1n3ja  1hks_  1bkra  1k42a  1dcja  1vig_  1jiga  1ail_  1bd8_  1bbpa  1k7ha  1hq0a  1ygha  1ajya  1ntca  1a56_  1jrma  1l6na  1qkfa  1kveb  1poa_  1mtyg  1f1ga  1m38b  1knla  1suh_  1iata  1adr_  1fi5a  1lk5a  1cx1a  1fsha  1vie_  1k9ka  3wrp_  1p1ma  1iu8a  1pbv_  1de1a  1ab3_  1pbwa  119l_  1cdb_  1icja  1ljpa  1f3ya  1d7qa  1atzb  1ny9a  1ic6a  1daqa  1qhqa  1tvt_  1gxmb  1jyga  1kwga  1hqi_  1iq6b  1jfxa  1liza  1i71a  1g4fa  2acy_  7ahlb  1jbia  1f1ma  1irl_  1dk0a  1owta  1mkaa  1e5ui  1ie5a  1d0qa  1f2tb  1chma  1i1sa  1esl_  1ewia  1nkr_  1ds9a  1yub_  1nbaa  1bpr_  1kria  1guia  1oboa  1beba  1ge9a  1jdw_  3thia  1b8pa  1jfra  1e6ye  1gr3a  1d7pm  1ctf_  1c99a  1ocp_  1tsg_  1rfa_  1ibya  1ew4a  1sll_  1dlwa  1cz1a  1d8ba  1ocka  1iqoa  1g3ga  1ddba  1p4ka  1jjfa  1iz6a  1a63_  1bxya  1fvl_  1eqka  1hx2a  1nlbl  1j57a  1ae3_  2jhba  1el4a  1k3ka  1jfub  1k0sa  1pou_  1wwca  1jboa  1chd_  2ezm_  1cz4a  1c02a  1fce_  1kv9a  1nsj_  1wab_  1ajw_  1n1jb  1rxr_  1dupa  1cnza | 1d4ba  1axn_  1neq_  1jf9a  1ghha  1lwda  1fxla  1h8ca  1fura  1qoya  1gm0a  1n3ka  1jcua  3lria  1edg_  1hhna  1il6_  2pii_  1amx_  1ihga  1aoy_  1b22a  1bta_  1dmo_  1qtoa  1kjs_  1c3ya  1e88a  1kh0b  1ah1_  1ijca  1eur_  1eo0a  1ib2a  1fhs_  1i42a  1eu8a  1af8_  1ge7a  1ap0_  1tif_  1ezta  1g9la  1mtpa  1c7ka  1byra  1lr5a  1ga3a  1gd5a  1bf2_  1bby_  1k2ea  1bgva  1yme_  1bd3a  1k7ia  2kauc  1m5za  1sgg_  4pgaa  1eswa  2bida  1nox_  1d1za  1bak_  1vls_  1pfsa  1fi2a  1i17a  1pnj_  1dbs_  1m3va  1jyta  1cdca  1gyha  2eboa  1bpv_  1odkd  1hkya  1ngr_  1ezga  1j8ca  1ekga  1m0zb  1gdva  1dpsd  1bjx_  1d1ra  1ag4_  1qk9a  1erza  1h4ya  1jw2a  1mwpa  1me3a  1aa3_  1hufa  1buya  1ld8a  1i2ta  1dqza  1d8ja  1i1na  1coua  1my5b  1j8ia  1iw5a  1mxra  1mnta  1m2ea  1jr6a  1nfa_  1dora  1oaja  1ltua  1aj3_  1coza  1akz_  2kinb  1j6ya  1i4va  1jr5a  1h4ib  1qm9a  1nhp_  1evxa  1qrjb  1lmja  1ikta  1mg4a  1imt_  1sro_  1dv5a  1k8ha  1ld8b  1ozna  1tvxb  1c9fa  1ayoa  1g2ha  1qisa  1bf6b  1c52_  3lip_  1bisb  1euaa  1l9fb  1whi_  1fyba  1jm7a  1ah9_  1eptb  1f3ca  1mvya  1br0a  1amm_  1btn_  1svq_  1g47a  1i4ua  1u2fa  1bwma  1fna_  1cpq_  1ip9a  1m9za  1k0mb  2bbkh  1aru_  1h4ra  1hfh_  1in1a  1kppa  1adn_  1tca_  1dgua  1ooea  1tbn_  1jb3a  1qr9a  1hxva  1ll8a  1fnc_  1c5ea  1uae_  1jfja  1bhe_  1flma  1eo9b  3mbp_  2sxl_  1qsva  1tl2a  1l2ma  1hzma  1apj_  2fha_  1ad2_  3crd_  1kr7a  1yua_  1guda  1qfta  1hoe_  1hp8_  1jli_  1kp6a  1c9oa  2bbkl  1ghc_  1bdc_  1eyha  2u1a_  1hn6a  1f53a  1esxa  1apya  1vsra  1k1ea  1o6xa  1kmda  1klpa  1ku8a  1k94a  1rdo2  1amp_  1c1fa  1htrb  1n6za  1khia  1gd1o  1lv3a  1cfe_  1jbaa  1mola  1a12a  1lq7a  1ci9a  1vfra  1ep0a  1gnd_  1ku0a  1psza  1lki_  1c8pa  1b1a_  1lfup  1mm4a  1coo_  1exka  1n72a  1ew6a  1loua  1fl2a  1i6za  1ubi_  1n9da  1pnbb  1coka  1m58a  1gn0a  1g84a  1g0sb  1e4ua  1ddf_  1eo9a  1fm4a  1gyfa  1egxa  1stu_  1k6wa  2alca  1b4ra  1ejba  1cjca  1lmza  1pama  1nkl_  1h67a  1jxca  1ecsa  1o6wa  2tgi_  1a5r_  1iml_  1a28b  1xnaa  1l2tb  1ml4a  1gk2a  1n9ja  256ba  1svpa  1hyp_  1ayx_  1cto_  1g7da  1bci_  1nsoa  1jr2b  1jd5a  1bno_  1rmg_  1vfya  1cv8_  1i6pa  1jjga  1mqkh  1qgpa  1uok_  1j7ga  1gpea  1bct_  1opka  1h63a |
